# Revision rates after pediatric PITA: A comparative study of microdebrider and coblation techniques

**DOI:** 10.1007/s00405-026-10421-7

**Published:** 2026-07-14

**Authors:** Nabil Zahalka, Sholem Hack, Malek Younis, Avia Carmon, Rotem Elul, Firas kassem, Ameen Biadsee

**Affiliations:** 1 Department of Otolaryngology-Head and Neck Surgery, Meir Medical Center, Kfar-Saba, Israel; 2 Gray Faculty of Medical and Health Sciences, Tel-Aviv University, Tel-Aviv, Israel; 3 City St. George’s University London School of Medicine, Program Delivered by University of Nicosia at the Chaim Sheba Medical Center, Ramat Gan, Israel

**Keywords:** Tonsillectomy, Intracapsular, Adenoidectomy, Coblation, Microdebrider, Obstructive Sleep Apnea, Pediatric, Reoperation, Treatment Outcomes

## Abstract

**Purpose:**

To compare long-term revision rates after pediatric partial intracapsular tonsillectomy and adenoidectomy (PITA) performed using microdebrider versus coblation techniques, and to evaluate whether operative method was independently associated with revision risk.

**Methods:**

This retrospective cohort study included healthy children younger than 16 years who underwent PITA for obstructive sleep-disordered breathing, 2016, when coblation technology was introduced and 2023, at a tertiary medical center. Intracapsular reduction of palatine tonsils and adenoids was performed using either powered microdebrider or coblation. The primary outcome was revision PITA for recurrent obstructive symptoms. Revision-free survival was analyzed using Kaplan-Meier methods and compared with log-rank testing. A multivariable Cox proportional hazards model adjusted for age and sex was used. Tonsil size was evaluated as a secondary covariate.

**Results:**

A total of 827 children were included (microdebrider, *n* = 478; coblation, *n* = 349). Median follow-up was 60 months overall (60 vs. 47 months). Revision occurred in 31 children: 6 (1.4%) in the microdebrider group and 25 (10.1%) in the coblation group. After 36 months, revision-free survival was 99.4% for microdebrider and 96.1% for coblation (log-rank *p* < 0.001). Coblation was independently associated with increased revision risk (HR 7.17; 95% CI 2.92–17.60; *p* < 0.001). Age, sex, and tonsil size were not significant predictors.

**Conclusion:**

In this institutional cohort, microdebrider PITA was associated with lower long-term revision rates than was coblation during the period of coblation was introduced. These findings highlight the influence of institutional experience and procedural execution on revision outcomes after intracapsular surgery and should not be interpreted as evidence of intrinsic device superiority.

## Introduction

Obstructive sleep-disordered breathing is one of the most common indications for adenotonsillar surgery in children. Intracapsular tonsillectomy has gained increasing popularity as a tissue-preserving alternative to the traditional extracapsular approach [1–4]. Partial intracapsular tonsillectomy and adenoidectomy (PITA) has been shown to reduce postoperative pain, accelerate return to normal diet and activity, and lower the risk of postoperative hemorrhage compared with total tonsillectomy, making it an appealing option for the management of pediatric obstructive symptoms [5–7]. Several instrument platforms are available for intracapsular resection, with microdebrider- and coblation-based techniques representing the two most widely used modalities [8–10]. 

Despite their widespread use, important uncertainties remain regarding the long-term revision outcomes of different intracapsular techniques. Large retrospective series have generally demonstrated low reoperation rates after powered microdebrider tonsillotomy, typically ranging from 1% to 4% over several years of follow-up [11–15]. In contrast, studies evaluating coblation intracapsular tonsillectomy have reported more variable revision rates, with some centers observing excellent long-term outcomes and others noting higher frequencies of regrowth during the early phases of adopting the technique [12, 16–18]. This variability has raised questions about whether differences between methods reflect intrinsic device performance, the nuances of procedural execution, or the influence of operator experience [10, 18–20]. 

Improved clarity regarding the relative durability of microdebrider versus coblation intracapsular tonsillectomy is clinically relevant, as recurrence of obstructive symptoms may necessitate revision surgery with increased operative morbidity [11, 12, 14, 18, 21]. Robust comparative data with extended follow-up are limited, and few studies have directly evaluated technique-specific revision rates within the same institution, where patient selection, perioperative care, and follow-up patterns are consistent.

The present study examined long-term outcomes after PITA performed at a single tertiary medical center, using either the microdebrider or coblation technique, in a large pediatric cohort. The primary objective was to compare revision surgery rates between techniques over a multi-year follow-up, with the goal of better defining the relative durability of each method in routine clinical practice. The specific hypothesis was that operative technique would remain associated with revision after accounting for baseline demographic and anatomic surrogates available in the medical record, including tonsil size.

## Methods

This retrospective cohort study was conducted at a tertiary medical center and included all pediatric patients who underwent PITA for obstructive sleep-disordered breathing January 1, 2016 to December 31, 2023. Coblation technology was introduced into departmental practice at the beginning of the study period; therefore, the timeframe was selected to capture the earliest coblation cases and ensure adequate follow-up for revision analysis.

### Patient selection and indications

Inclusion criteria were age younger than 16 years at the time of surgery, symptoms consistent with obstructive sleep apnea, and underwent combined intracapsular reduction of both the palatine tonsils and adenoids. In accordance with the 2019 American Academy of Otolaryngology–Head and Neck Surgery guidelines and widely used pediatric adenotonsillectomy practice patterns, polysomnography was not routinely required for otherwise healthy children [22]. 

Surgical indication was based on clinical evaluation for obstructive sleep-disordered breathing (e.g., habitual snoring, witnessed apneas, labored breathing during sleep, daytime behavioral symptoms) together with adenotonsillar hypertrophy on examination [23–25]. Children with neuromuscular disorders, genetic syndromes, storage diseases, severe obesity (body mass index for age at the 99th percentile or above), craniofacial abnormalities or systemic comorbidities known to affect airway tone were excluded. Children with prior adenotonsillar surgery were also excluded. Procedures limited to adenoidectomy alone or tonsillotomy alone were also excluded to maintain a uniform surgical cohort.

### Surgical technique

In the microdebrider group, adenoidectomy and intracapsular tonsillar reduction were performed using a StraightShot M5 microdebrider handpiece (Medtronic Xomed, Inc., Jacksonville, FL, USA) connected to a Medtronic Integrated Power Console System. A Tricut blade (11 cm × 4 mm) was used for both the tonsil and adenoid procedures. The microdebrider was set at 3000 rpm, and the tonsils were reduced to the capsule under direct visualization, leaving a thin residual tonsillar cuff with preservation of the capsule. Hemostasis was achieved using monopolar suction diathermy at 25 W for both the tonsillar bed and the adenoid bed.

In the coblation group, intracapsular tonsillar reduction and adenoid reduction were performed using the Coblator II system with an RF 8000E wand (ArthroCare Corp., Austin, TX, USA). According to departmental protocol, the ablation was set at 7 and the coagulation at 3. For both techniques, the intended endpoint was intracapsular reduction leaving a thin residual tonsillar cuff over the capsule, with preservation of the capsule. All procedures were performed by otolaryngology residents under the direct supervision of a fellowship-trained pediatric otolaryngologist. Technique allocation reflected departmental practice patterns during the study period (including staged introduction of coblation) rather than patient-level randomization.

Patients were divided into two groups based on operative technique. Demographic variables, tonsil size, operative notes, and follow-up encounters were extracted from the electronic medical records. Cases were identified via an institutional electronic medical record query of operative logs using procedure codes and standardized operative note terminology for intracapsular tonsil and adenoid reduction over the specified timeframe, followed by manual verification of operative technique and patient eligibility. Preoperative tonsil size was recorded using the Brodsky Tonsillar Grading Scale documented in the preoperative assessment (grades 1–4). For analysis, grades 1–2 were combined in secondary models due to low event counts in grade 1.

The primary outcome was revision surgery, defined as a subsequent PITA involving both palatine tonsils and adenoid tissue for recurrent obstructive symptoms. Revision procedures limited to tonsils-only or adenoid-only were excluded. This definition was used to maintain a uniform endpoint representing a clinically significant recurrence requiring repeat comprehensive adenotonsillar intervention, and to avoid heterogeneity introduced by isolated adenoid or tonsil procedures performed for distinct indications.

Follow-up duration was calculated from the index operation to either revision or the last documented clinical encounter. Revision indications were determined by a fellowship-trained pediatric otolaryngologist who was the head of the Pediatric Otolaryngology Division throughout the study period, ensuring consistent application of revision thresholds across both surgical technique groups. The clinical trigger for revision was the recurrence of obstructive sleep-disordered breathing symptoms (habitual snoring, witnessed apneas, restless sleep, or labored breathing) after an initial symptom-free interval following the index PITA. Children with recurrent symptoms underwent comprehensive evaluation in the pediatric otolaryngology clinic, including detailed history, oral examination, and fiberoptic nasopharyngoscopy to confirm tonsillar and adenoid regrowth and to exclude alternative contributing factors (e.g., allergic rhinitis, nasal obstruction, weight gain). Polysomnography was obtained when feasible; however, because pediatric polysomnography is not available at our institution and access through the national health system involves long waiting periods, only 23 children underwent confirmatory polysomnography prior to revision. For the remaining children, the indication was based on the consistent constellation of recurrent obstructive symptoms together with objective evidence of adenotonsillar regrowth on examination. Revisions performed for non-obstructive indications (e.g., recurrent infections, peritonsillar abscess) were not included in the primary endpoint. Revision procedures were performed using either microdebrider or coblation, with the technique selected at the discretion of the operating surgeon at the time of revision.

### Statistical analysis

Categorical variables were summarized as frequency and percentage. Gender was compared between surgical technique groups using the chi-square test. Age distribution was assessed using histograms and compared using the Kruskal-Wallis test, with Dunn’s post hoc procedure and Bonferroni correction applied for multiple comparisons. Median follow-up was estimated using the reverse-censoring method. Tonsil size grade distributions were compared between technique groups using a Mann-Whitney U test and contingency tables.

Revision-free survival was evaluated using Kaplan–Meier analysis, with comparisons between surgical techniques performed using a log-rank test. To assess the independent association between operative method and revision risk, a multivariable Cox proportional hazards model was constructed, adjusting for age at surgery and sex. Secondary models evaluated tonsil size as a covariate to address baseline anatomic confounding. All statistical tests were two-sided, with significance set at *p* < 0.05. Analyses were performed using SPSS version 29 (IBM Corp., Armonk, NY, USA).

## Results

A total of 827 children met the study criteria and underwent PITA between 2016 and 2023. Of these, 478 (57.8%) were treated with the microdebrider technique and 349 (42.2%) with coblation. The cohort included 491 males (59.3%) and 336 females (40.7%). The mean age at surgery was 58.6 months (95% CI, 58.1–59.1). Age distributions were similar between groups, with a mean of 59.5 months (95% CI, 59.1–59.9) in the microdebrider group and 57.2 months (95% CI, 56.1–58.4) in the coblation group. The age range across the cohort was 8 to 222 months. No statistically significant demographic differences were identified between groups (Table 1).

**Table 1:** Baseline characteristics of children undergoing partial intracapsular tonsillectomy and adenoidectomy (PITA)

Characteristic	Overall	Microdebrider	Coblation
Mean age (months, 95% CI)	58.6 (58.1–59.1)	59.5 (59.1–59.9)	57.2 (56.1–58.4)
Age range (months)	8–222	—	—
Female, n (%)	336 (40.6%)	193 (40.4%)	143 (41.0%)
Male, n (%)	491 (59.4%)	285 (59.6%)	206 (59.0%)
Microdebrider, n (%)	478 (57.8%)	—	—
Coblation, n (%)	349 (42.2%)	—	—
Median follow-up (months)	60	60	47
≥ 24 months follow-up, n (%)	729 (88.1%)	446 (93%)	283 (81.1%)
Tonsil size grade 1, n (%)	4 (0.5%)	3 (0.6%)	1 (0.3%)
Tonsil size grade 2, n (%)	184 (22.2%)	111 (23.2%)	73 (20.9%)
Tonsil size grade 3, n (%)	435 (52.6%)	248 (51.9%)	187 (53.6%)
Tonsil size grade 4, n (%)	204 (24.7%)	116 (24.3%)	88 (25.2%)
Tonsil size distribution p-value	—	—	0.451

Values are presented as mean (95% confidence interval), number (percentage), or median (range), as appropriate. Follow-up duration reflects time from index operation to last clinical encounter. There were no significant differences in age or sex distribution between groups

Postoperative follow-up was available for all patients. The median follow-up duration for the entire cohort was 60 months. Median follow-up was 60 months in the microdebrider group and 47 months in the coblation group. A total of 729 patients (88%) had at least two years of follow-up, including 446 (93%) in the microdebrider group and 283 (81.1%) in the coblation group (*p* < 0.001).

**Fig. 1:**
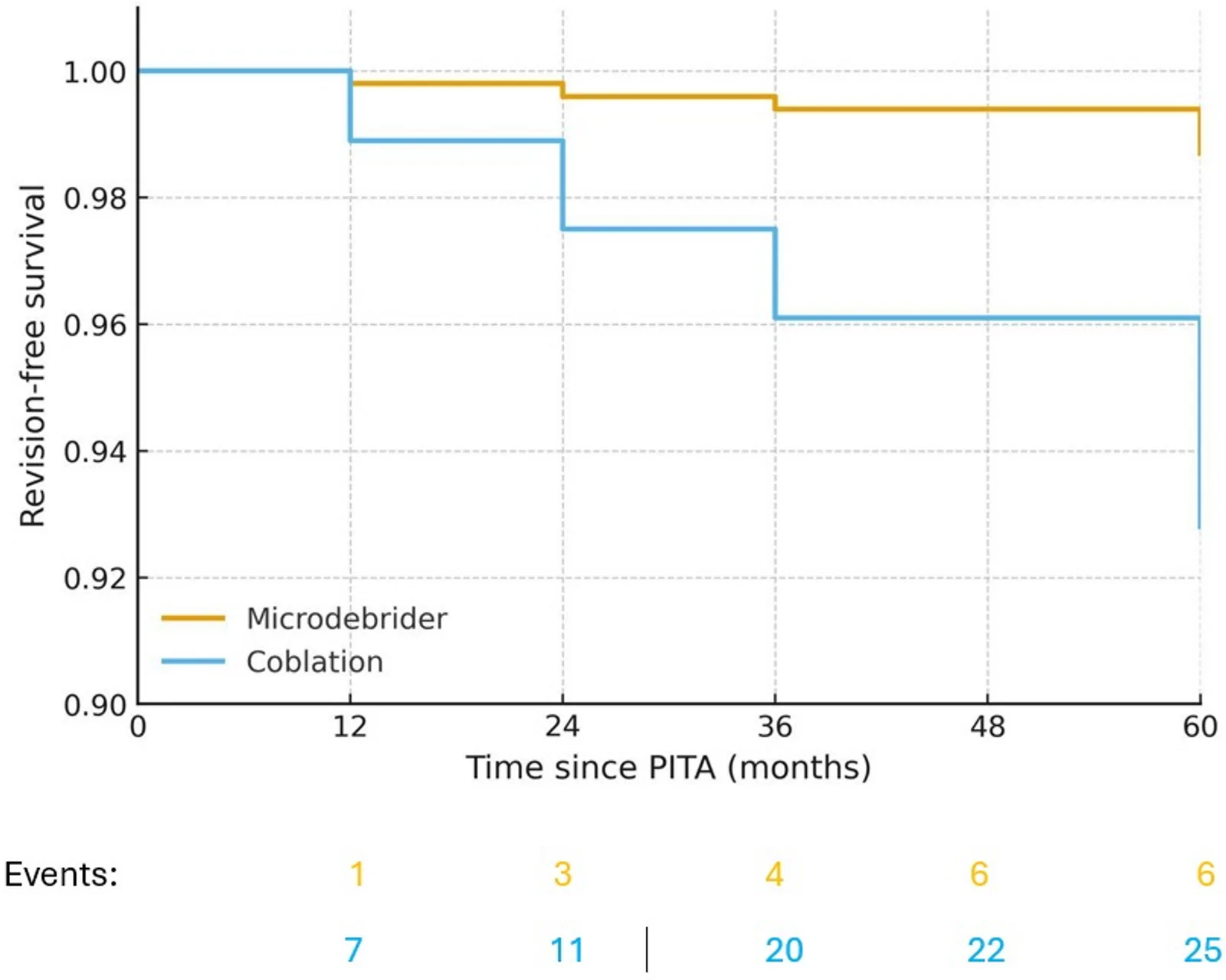
Kaplan-Meier revision-free survival Kaplan-Meier curves comparing revision-free survival after pediatric PITA by surgical technique. The microdebrider group had longer survival than coblation at 12–36 months (log-rank p < 0.001)

Revision surgery occurred in 31 children during the study period; 25 in the coblation group (10.1%), and s6 in the microdebrider group (1.4%). Time to revision ranged from 6 to 47 months in the microdebrider group and from 8 to 60 months in the coblation group. The cumulative revision-free survival proportions at 12, 24, and 36 months were 99.8%, 99.6%, and 99.4% in the microdebrider group, and 98.9%, 97.5%, and 96.1% in the coblation group (Table 2). Kaplan-Meier analysis showed a significant difference in revision-free survival between groups on log-rank testing (*p* < 0.001). Median time to revision was not reached in either group (Table 2; Fig. 1).

**Table 2:** Revision outcomes and revision-free survival following pediatric PITA

Outcome	Microdebrider	Coblation	Total
Revision PITA for recurrent obstruction, n (%)	6 (1.4%)	25 (10.1%)	31 (3.7%)
Time to revision (months)	6–47	8–60	6–60
Revision-free survival, 12 months (%)	99.8%	98.9%	—
Revision-free survival, 24 months (%)	99.6%	97.5%	—
Revision-free survival, 36 months (%)	99.4%	96.1%	—

Revision surgery was defined as repeat intracapsular resection of both palatine tonsils and adenoids for recurrent obstructive symptoms

Time to revision is reported as the range in months from the index surgery to revision

Revision-free survival percentages were derived from Kaplan-Meier estimates at fixed timepoints of 12, 24, and 36 months

**Fig. 2:**
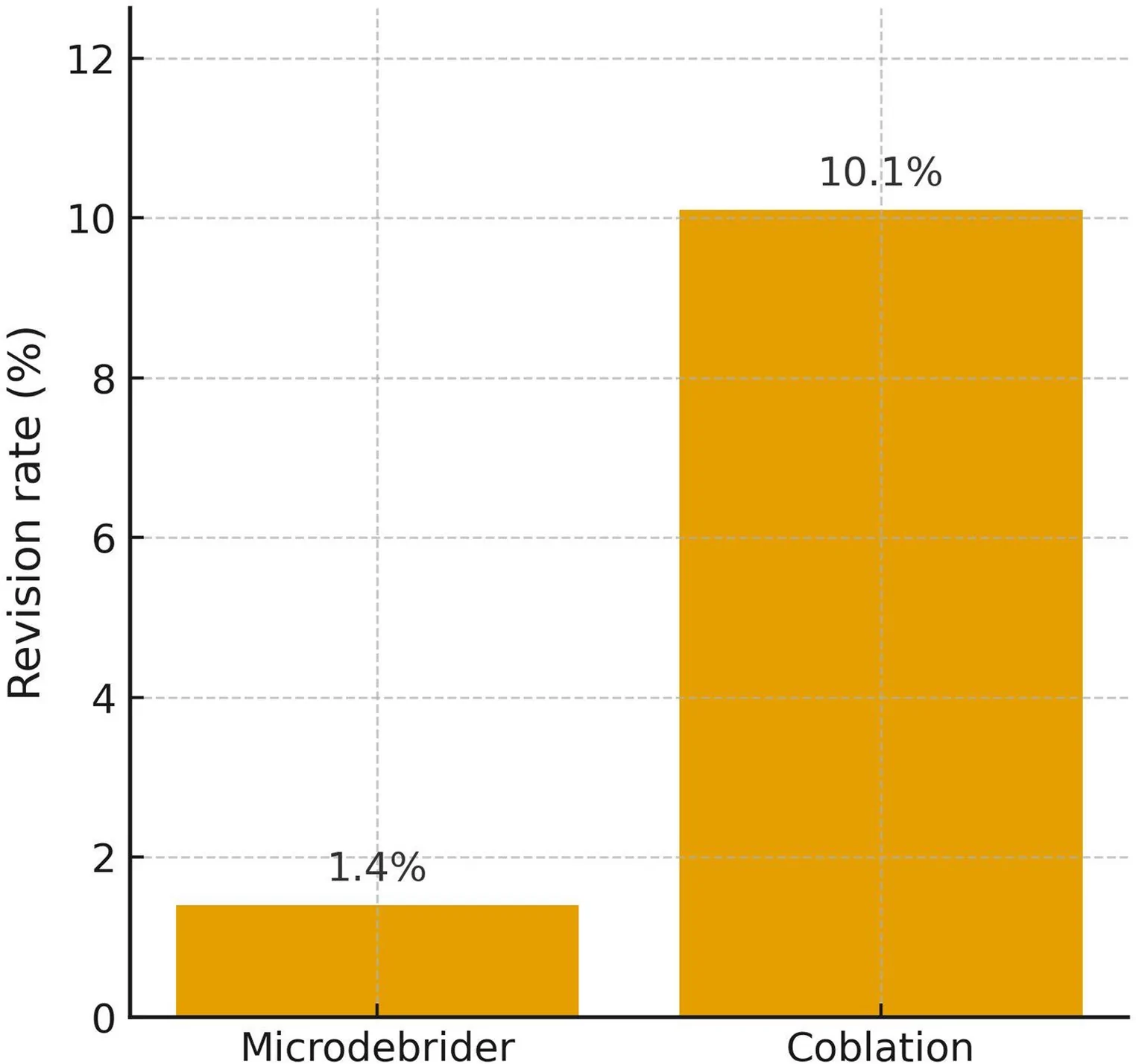
Revision rates by surgical technique Proportion of pediatric PITA patients requiring revision surgery, stratified by technique. Revisions occurred in 1.4% of microdebrider cases and 10.1% of coblation cases

The absolute difference in revision probability is illustrated by the proportion of children undergoing repeat PITA—1.4% in the microdebrider group versus 10.1% in the coblation group (Fig. 2).

**Fig. 3:**
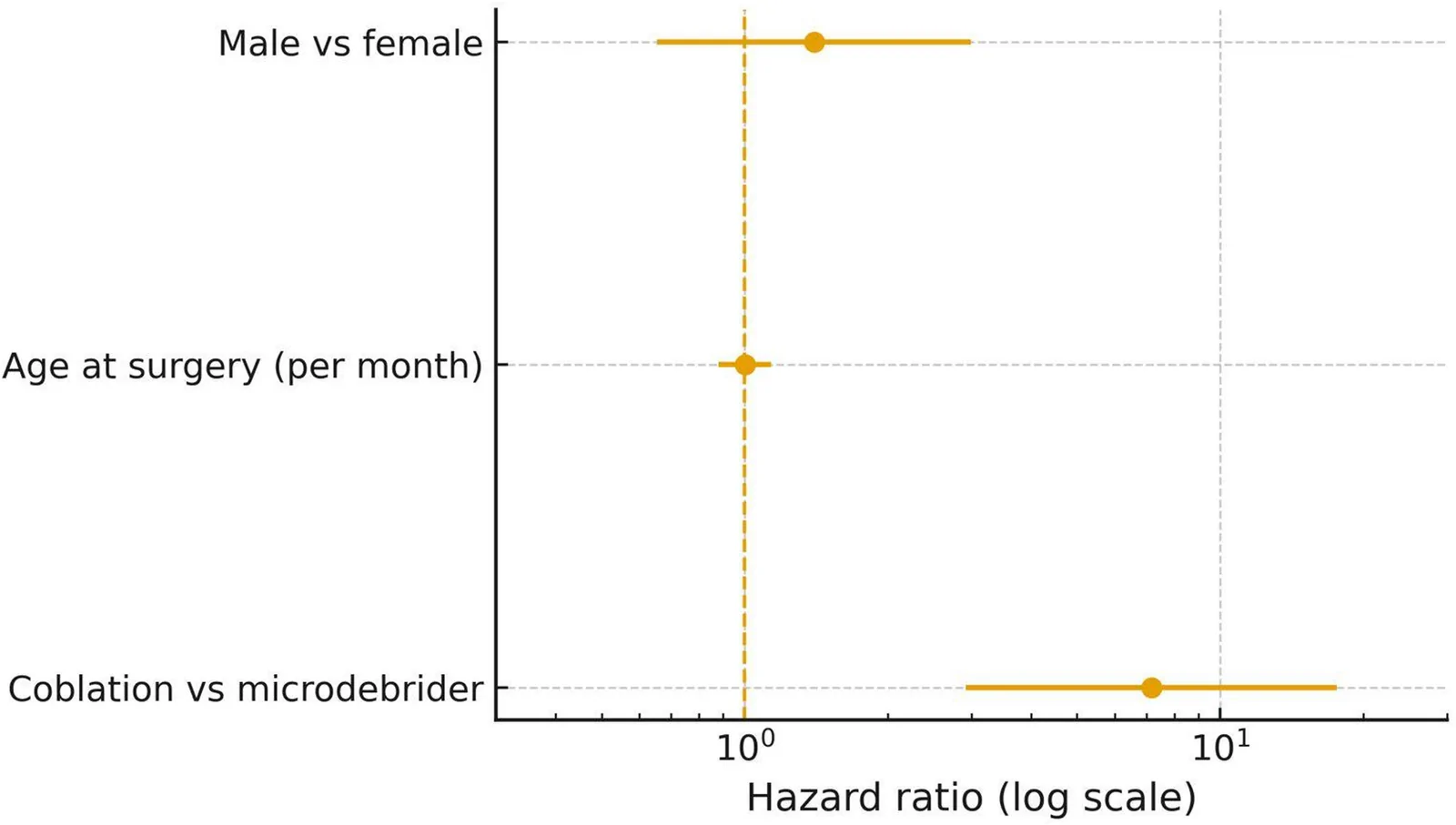
Multivariable Cox model for revision risk Forest plot of Cox model for revision after pediatric PITA. Coblation showed increased hazard risk for revision versus microdebrider (HR 7.17; 95% CI 2.92–17.60)

On multivariable Cox proportional hazards analysis, coblation was associated with a greater hazard for revision compared with the microdebrider technique (HR 7.168; 95% CI 2.919–17.601; *p* < 0.001). Age at surgery (HR 1.002; 95% CI 0.882–1.137; *p* = 0.981) and sex (HR 1.399; 95% CI 0.654–2.991; *p* = 0.386) were not associated with revision risk. Proportional hazards assumptions were met for all variables (Table 3; Fig. 3).

**Table 3:** Multivariable Cox Proportional Hazards model for risk of revision surgery

Variable	HR	95% CI	*p*-value
Coblation vs. microdebrider	7.168	2.919–17.601	< 0.001
Age at surgery (per month)	1.002	0.882–1.137	0.981
Male vs. female	1.399	0.654–2.991	0.386

Hazard ratio (*HR*) > 1.0 indicate increased risk for revision

The model was adjusted for age at surgery and for sex

Coblation technique was independently associated with greater revision risk compared with microdebrider

Age and sex were not significant predictors in the adjusted model

Proportional hazards assumptions were satisfied for all variables

Tonsil size grading in the preoperative assessment did not differ significantly between surgical technique groups (Mann-Whitney U test, *p* = 0.451). The distribution across tonsil grades was similar between microdebrider and coblation cohorts (Table 1). In secondary models, tonsil size was not independently associated with revision and did not materially change the association between technique and revision risk.

No additional demographic or operative variables differed between patients who underwent revision surgery and those who did not.

A sensitivity analysis evaluated isolated tonsil-only and adenoid-only revisions that were not included in the primary endpoint. Eleven children underwent isolated revision adenoidectomy (3 in the microdebrider group, 8 in the coblation group), and 6 underwent isolated revision tonsillotomy (1 in the microdebrider group, 5 in the coblation group). When these 17 cases were added to the primary endpoint as a combined any-revision endpoint, the total counts were 10/478 in the microdebrider group and 38/349 in the coblation group. The multivariable Cox proportional hazards model using this expanded endpoint yielded a HR of 6.50 (95% CI 3.22–13.12; *p* < 0.001) for coblation versus microdebrider, confirming that the magnitude, direction, and statistical significance of the between-group differences were preserved when isolated revisions were included. Bootstrap simulation (1,000 iterations) confirmed statistical significance under the expanded endpoint definition.

To address whether the technique-related difference in revision risk varied by age, a stratified analysis was performed across three age categories: <3 years (*n* = 248), 3 to < 6 years (*n* = 400), and ≥ 6 to 15 years (*n* = 179). Coblation was associated with a higher revision rate than microdebrider in every age subgroup: 4.2% versus 1.6% (HR 2.80, 95% CI 0.54–14.45) in children < 3 years; 9.6% versus 0.8% (HR 13.53, 95% CI 3.09–59.25) in children 3 to < 6 years; and 6.9% versus 1.9% (HR 6.10, 95% CI 1.18–31.62) in children 6 to 15 years A formal test for interaction between age and surgical technique was non-significant (*p* = 0.78), indicating that the magnitude of the technique-related effect did not vary significantly across age strata (Table 4).

**Table 4:** Revision outcomes stratified by age subgroup

Age subgroup	*n*	Micro. *n* (%)	Cob. *n* (%)	HR (cob. vs. micro.)	95% CI	*p*-value
< 3 years	248	2/128 (1.6%)	5/120 (4.2%)	2.80	0.54–14.45	0.22
3 to < 6 years	400	2/243 (0.8%)	15/157 (9.6%)	13.53	3.09–59.25	< 0.001
≥ 6 to 15 years	179	2/107 (1.9%)	5/72 (6.9%)	6.10	1.18–31.62	0.031

*Micro* microdebrider, *Cob* coblation

Hazard ratios derived from technique-only Cox proportional hazards models within each age group. A formal interaction test between age and surgical technique was non-significant (*p* = 0.78), indicating that the relative effect of technique on revision risk did not differ significantly across age strata. Total cohort across the three subgroups: *n* = 827

## Discussion

The results of this study indicated a substantial difference in revision surgery rates between two commonly used pediatric intracapsular adenotonsillectomy techniques over an extended period. Over a median follow-up of approximately 5 years, children treated with microdebrider resection demonstrated a persistently lower revision rate, while those treated with coblation, during its early adoption, had an elevated risk for revision surgery. This difference remained significant across both Kaplan-Meier survival analyses and multivariable adjustments for age and sex, suggesting that procedural execution and residual tissue burden may contribute to long-term revision outcomes. Importantly, baseline tonsil size grading was similar between cohorts and was not independently associated with revision, supporting that the observed difference was not likely due to gross differences in preoperative tonsillar hypertrophy.

The low revision rate associated with microdebrider intracapsular tonsillectomy aligns with large, multicenter retrospective series reporting reoperation incidences of 1% to 4% over extended follow-up periods [17, 26, 27]. For example, a 15-year retrospective review of over 12,000 pediatric intracapsular tonsillectomies recorded a revision rate of 1.39% with a mean time to revision of 3.5 years [26]. These findings suggest that when intracapsular tissue reduction is performed thoroughly and consistently, powered intracapsular resection may provide durable long-term control of obstructive symptoms in appropriately selected children.

The elevated revision rate observed in the coblation cohort should be interpreted in the context of published outcomes. A recent large pediatric tertiary-center study of 4,111 coblation intracapsular tonsillectomies reported an overall revision rate of 3.3% over 9 years of follow-up [18]. Other series reported revision rates ranging from approximately 1% to over 10%, with variability influenced by follow-up duration, patient age distribution, and institutional practice patterns [28]. The observed rate reported in the present study lies at the upper end of this spectrum. The differences across series suggest that long-term revision outcomes after intracapsular techniques may be influenced by procedural execution, surgeon experience, and the extent of residual lymphoid tissue following surgery, underscoring the importance of longitudinal, institutional outcome tracking when comparing surgical platforms.

Several factors may explain the difference in revision rates observed between the two intracapsular techniques. Microdebrider tonsillotomy provides a clear anatomical endpoint as tissue is shaved down to the tonsillar capsule, and any residual lymphoid tissue is visibly removed [13]. In addition, the use of suction diathermy for hemostasis after microdebrider resection may further reduce the likelihood of leaving small islands of deeper lymphoid tissue near the capsule. In contrast, coblation reduces tonsillar tissue volume while simultaneously providing hemostasis, typically leaving a superficial coagulated layer on the tonsillar bed. This eschar can partially obscure the underlying capsule, raising concern that small islands of deeper lymphoid tissue may persist if not carefully sought and ablated [12, 29]. This “visual-feedback gap” can lead to under-resection during early use, potentially contributing to recurrent obstructive symptoms and later revision, a phenomenon consistent with expert caution about coblation’s deceptive visual endpoints [30]. The present findings, together with published experience, suggest that differences in procedural execution and residual tissue burden may contribute to long-term revision outcomes independent of device platform alone.

An important contextual factor in interpreting these findings is that coblation was introduced into departmental practice at the start of the study period, meaning that a substantial proportion of coblation cases were performed during the early institutional adoption phase. The learning curve associated with coblation intracapsular tonsillectomy is well-recognized. The technique’s simultaneous ablation and hemostasis create a visual endpoint that differs fundamentally from the mechanical tissue removal of the microdebrider, and early operators may underestimate residual tissue burden beneath the coagulated eschar. Published series have demonstrated that revision rates with coblation decline meaningfully as institutional and individual surgeon experience accumulates [18, 27]. It is therefore plausible that a portion of the elevated revision risk observed in our coblation cohort reflects technique-specific, early-adoption effects rather than intrinsic device inferiority. This temporal confound could not be fully adjusted for in the current analysis, and the observed hazard ratio should be interpreted with this in mind.

The differential follow-up between microdebrider and coblation groups (median 60 vs. 47 months, respectively) reflects the staged introduction of coblation at our institution rather than differential loss to follow-up. Importantly, this imbalance presents a bias against the observed effect: the microdebrider cohort had a longer at-risk window in which late revisions could accrue, yet exhibited substantially fewer events. Kaplan-Meier and Cox analyses accounted for unequal follow-up through censoring, and the survival curves diverged within the first 12 to 24 months — well before the point at which the follow-up windows of the two cohorts began to differ materially. Time to revision in the coblation group ranged from 8 to 60 months, indicating that revisions occurred throughout the available follow-up window rather than clustering at the late end where microdebrider follow-up extended further than coblation. Nevertheless, late revisions in the coblation cohort beyond the current follow-up window cannot be excluded, and continued surveillance is warranted to capture any late events.

An age-stratified analysis was performed across three categories (< 3 years, 3 to < 6 years, and ≥ 6 to 15 years). The technique-related difference in revision risk persisted across all age subgroups, with coblation associated with higher revision rates than microdebrider in children younger than 3 years (4.2% vs. 1.6%), 3 to < 6 years (9.6% vs. 0.8%), and 6 to 15 years (6.9% vs. 1.9%). A formal test for interactions between age and surgical technique was not significant (*p* = 0.78), indicating that the relative effect of technique on revision risk was consistent across the pediatric age spectrum. Whereas prior series identified younger age (particularly under 2–4 years) as a risk factor for regrowth and reoperation, our data did not reproduce this pattern: microdebrider revision rates were uniformly low (0.8–1.9%) across all age groups, suggesting that thorough intracapsular tissue reduction may reduce the likelihood of recurrent obstructive symptoms across age groups [12, 18, 26]. Similarly, the absence of sex differences was consistent with prior observations that regrowth is tissue- and technique-dependent rather than influenced by gender.

From a clinical decision-making perspective, the difference in reoperation probability between techniques is meaningful. Even though absolute revision rates remain modest, a near seven-fold relative increase in reoperation risk with coblation cannot be ignored, particularly because revision surgery in our cohort consisted of a second intracapsular procedure, whereas some centers may convert to extracapsular dissection with higher associated morbidity. In settings where long-term control of airway obstruction is a priority, these findings suggest potential differences in revision outcomes that warrant prospective evaluation. In contrast, coblation outcomes can vary between centers for several reasons, including differences in technique, equipment handling, and tissue-ablation characteristics. On the other hand, the literature indicates that with sufficient experience and refined technique, coblation can achieve revision rates comparable to those of microdebrider methods [18, 27]. Therefore, technique selection should balance short-term perioperative morbidity considerations (including postoperative pain, recovery profile, and risk of hemorrhage) with long-term revision outcomes, while also accounting for institutional experience, surgeon training, and procedural standardization.

It should be noted that revision risk represents only one dimension of outcome assessment after intracapsular surgery. Intracapsular techniques have been adopted widely, largely because of their favorable perioperative morbidity profile compared with extracapsular tonsillectomy, including reduced postoperative pain, earlier return to diet and activity, and lower rates of postoperative hemorrhage [5–9]. Accordingly, long-term revision outcomes should be interpreted alongside these short-term recovery advantages when comparing operative approaches. The present study was not designed to systematically evaluate postoperative bleeding, pain, or recovery outcomes between techniques, and these variables were not consistently available within the retrospective dataset. Future prospective comparative studies should therefore assess both long-term revision outcomes and perioperative morbidity endpoints in parallel to better define the overall clinical trade-offs associated with each technique.

An additional consideration is that revision surgery represents a clinically meaningful but imperfect surrogate for true biologic tonsillar or adenoidal regrowth. The decision to proceed with revision intervention may be influenced not only by recurrent lymphoid hypertrophy, but also by symptom severity, parental preference, institutional follow-up practices, surgeon judgment, and local thresholds for reoperation. Although all revision cases in the present cohort underwent standardized clinical reassessment with endoscopic evaluation confirming recurrent adenotonsillar tissue contributing to obstructive symptoms, objective quantification of regrowth was not performed systematically. Accordingly, the present findings should be interpreted as differences in revision outcomes within a defined institutional practice environment rather than definitive evidence of differential biologic regrowth between techniques.

## Limitations

Several methodological limitations should be acknowledged. First, the retrospective design relied on the accuracy and completeness of electronic medical record documentation, which may not capture subtle postoperative symptoms or care received outside the institution. Second, although follow-up duration was substantial and exceeded two years in most children, surveillance was not uniform across all patients, and late revisions beyond the follow-up window cannot be excluded. Third, surgeon-level variables, including individual case volumes, training exposure, and comfort with each technique were not analyzed, limiting the ability to quantify operator-specific contributions to revision risk. Fourth, the staged introduction of coblation during the study period meant that early coblation cases reflected an institutional learning-curve. Operative year and surgeon-level identifiers were not available for stratified analysis, and learning-curve effects cannot be disentangled from technique-specific effects in the current dataset. The elevated hazard ratio observed for coblation should therefore be interpreted as an institutional early-adoption outcome rather than evidence of intrinsic device or procedural inferiority. Fifth, while the primary endpoint was restricted to repeat combined PITA, isolated tonsil-only or adenoid-only revisions may reflect tissue-specific recurrence that did not meet the threshold for combined revision. The sensitivity analysis incorporating these events yielded consistent findings (HR 6.50; 95% CI 3.22–13.12). Finally, the study did not incorporate patient-centered or perioperative morbidity outcomes, such as postoperative pain scores, hemorrhage rates, recovery trajectories, or sleep-related quality-of-life metrics, which may provide additional context when balancing short-term morbidity against long-term revision outcomes. Although polysomnography and BMI were not routinely documented for all otherwise healthy children in this clinical cohort, we incorporated available anatomic surrogates (tonsil size grading) and found no evidence that baseline tonsil sizes differed by technique or explained revision risk. Residual confounding by unmeasured severity markers remains possible and should be addressed in prospective or propensity-matched studies.

## Future directions

Future research should prospectively evaluate intracapsular adenotonsillectomy techniques using standardized operative protocols, predefined revision criteria, and uniform follow-up intervals. Randomized or propensity-matched designs would facilitate more precise comparison of long-term revision outcomes while reducing institutional and surgeon-selection biases. Future studies should also incorporate objective assessment of recurrent adenotonsillar hypertrophy using validated endoscopic grading systems, symptom instruments, and polysomnography when feasible. Systematic recording of postoperative hemorrhage, pain, recovery trajectories, and quality-of-life outcomes would provide a more comprehensive understanding of the risks and benefits of perioperative morbidity compared to long-term revision risk. Incorporating surgeon experience, operative volume, and learning-curve analyses may further clarify technique-specific performance over time. Extended longitudinal surveillance beyond five years may also help define late recurrence patterns and optimize evidence-based technique selection in pediatric obstructive sleep-disordered breathing.

## Conclusion

In this single-institution cohort spanning a period of coblation adoption, microdebrider PITA was associated with lower revision rates than coblation. These findings likely reflect a combination of procedural execution, residual tissue burden, and the institutional learning curve associated with adopting a new intracapsular method, rather than intrinsic differences in device performance. Both techniques remained effective for the majority of children, and the present results underscore the importance of structured training, technique standardization, and longitudinal outcome tracking when implementing new operative modalities. Further prospective evaluation using standardized operative protocols, objective outcome measures, and longer-term surveillance is warranted to better define long-term revision outcomes and inform evidence-based decision-making in pediatric obstructive sleep-disordered breathing.

## Declarations

### Data Sharing

Upon Request From The Authors.
